# Reliability of quantifying the spatial distribution of fatty infiltration in lumbar paravertebral muscles using a new segmentation method for T1-weighted MRI

**DOI:** 10.1186/s12891-016-1090-z

**Published:** 2016-05-27

**Authors:** Áine Ni Mhuiris, Thomas Volken, James M. Elliott, Mark Hoggarth, Dino Samartzis, Rebecca J. Crawford

**Affiliations:** Centre for Health Sciences, School of Health Professions, Zurich University of Applied Sciences, Technikumstrasse 81, Postfach, CH-8401 Winterthur Switzerland; Feinberg School of Medicine, Northwestern University, Chicago, USA; School of Health and Rehabilitation Sciences, University of Queensland, Brisbane, Australia; McCormick School of Engineering, Northwestern University, Evanston, USA; Department of Orthopaedics and Traumatology, The University of Hong Kong, Pokfalum, Hong Kong, SAR China; Faculty of Health Professions, Curtin University, Perth, Australia

**Keywords:** Reliability, Paravertebral muscle, Fatty infiltration, T1-weighted MRI, Lumbar spine

## Abstract

**Background:**

To our knowledge, there are no methods allowing for quantification of the spatial distribution of lumbar paravertebral muscle fatty infiltration (FI) in the transverse plane. There is an increasing emphasis on muscle tissues as modifiable factors in lumbar spine health. Population datasets based on conventional T1-weighted (T1-W) magnetic resonance imaging (MRI) represent a valuable resource for examining all spinal tissues, and methods with reliability are needed. The aim of our study was to determine the reliability of a novel method quantifying lumbar paravertebral muscle fat content based on conventional T1-W MRI.

**Methods:**

Axial 3-Tesla T1W MRIs from ten adult subjects (3W, 7M; mean age 52.8 ± SD 7.2 years) were randomly selected from the large prospective cross-sectional Hong Kong Population-based Disc Degeneration Cohort study examining lumbar spine degeneration. The selected sample included subjects with mixed imaging-determined disc degeneration and low back pain history. Two raters with MRI lumbar paravertebral muscle analysis experience (R1 > 250 h and R2 > 1000 h) repeat-measured the image-set a week apart. Multifidus and erector spinae (spinalis, longissimus and iliocostalis) were manually outlined together on a single-slice from the inferior vertebral end-plates of L1 to L5 using a semi-automated, quartile-defining (Q1-4 (medial to lateral) and Q_mean_) MatLab-based programme. Bland-Altman plots and intra-class correlation coefficients (ICC) with 95 % confidence intervals (CI) describe intra- and inter-rater reliability according to lumbar level, quartile, and side, and combined level and quartile.

**Results:**

There was good intra- (ICC = 0.88; CI: 0.87–0.90) and inter-rater agreement (ICC = 0.82; CI: 0.80–0.84). Intra-rater values for Q_mean_ (ICC; CI) were higher at L5 (0.89; 0.79–0.94) than L1 (0.61; 0.37–0.78). Higher intra-rater values for L1-5 were shown at Q1 (0.93; 0.91–0.95) than Q3 (0.83; 0.78–0.87) or Q4 (0.81; 0.76–0.85), and on the right (0.91; 0.90–0.93) than left (0.85; 0.83–0.88). Similar observations were made for inter-rater values in terms of lumbar level and quartile, with no differences between sides shown.

**Conclusions:**

In our study of ten cases we demonstrate a reliable method to quantify the spatial distribution of fat content in lumbar paravertebral muscles based on T1W MRI. Understanding the geography of fat content in these muscles may offer additional insight in determining and improving spinal health. The clinical relevance and application of this method require testing across various populations to build on the early feasibility established in this study.

## Introduction

Low back pain (LBP) is the world’s most disabling disease [1]. With lifetime prevalence reported to be as high as 84 %, and a 1-year prevalence between 22–65 % [2], LBP is a common condition that is forecast to have a wider impact on society [3] alongside our ageing population [4]. The mounting burden of LBP has come despite increased availability of surgical and non-surgical interventions [5]. New strategies are necessary to mitigate the crippling economic, social, and personal impact of the condition [6], and muscles of the trunk and lumbar spine are receiving increased attention as modifiable structures with both prognostic and therapeutic potential.

Cross-sectional [7–10] and longitudinal studies [11] evaluating paravertebral muscle quality using MRI have shown a relationship between muscle fatty infiltration (FI) and LBP. However, inconsistent associations are also reported [12], and are confounded by normative age-related change [13, 14], degenerative features of the vertebrae and discs [8, 15], and spinal curvature [16, 17]. As such, the etiological significance of FI is unclear and investigations to better understand the influence of muscle fat content on spinal health are needed.

While research has shown that lumbar paravertebral muscles infiltrate with fat, a surprisingly modest literature describes whether there is a geographical propensity for fat to accumulate. In order to best direct clinically meaningful interventions, this knowledge seems crucial. Low lumbar levels have more muscle fat than upper levels [9, 12, 13], which coincides with the greatest muscle volume [13], and other degenerative spinal features [18]. However, as far as we are aware, no studies have examined the spatial distribution of lumbar paravertebral muscle FI in the transverse plane. This is surprising when neck pain and disability relates to the presence of FI in the most medial muscle tissues [19], and that an exercise intervention, albeit preliminary, directed at such, improved muscle morphology, pain and function [20].

The contemporary standard for evaluating size and structure of soft-aqueous tissues like skeletal muscle is chemical-shift MRI producing water- and fat-only images from multi-echo acquisitions [10, 21–23]. Excellent accuracy has been shown for manual segmentation based on these imaging techniques against spectroscopy [10] and histology [24], and for various neuromusculoskeletal conditions [21, 25] including LBP [10, 26]. However, large ongoing population-based studies often use conventional T1-W MRI [12, 18], which represent a data resource of immeasurable value that muscle investigators would benefit from accessing. As such, a reliable method for quantifying FI from conventional T1-W MRI is necessary before clinical translation is effectively realised.

The aim of our study was to determine the reliability of a novel semi-automated segmentation method enabling quantification of the spatial distribution of lumbar paravertebral FI from axial T1-W MRI. We intended our study to provide preliminary evidence for the feasibility of quantifying the geography of fat content in muscle tissues, which can then be employed in studies examining spinal health.

## Materials and methods

Axial 3-Tesla T1-W MRIs from ten adult subjects were randomly-selected from the large prospective cross-sectional Hong Kong Population-based Disc Degeneration Cohort study undertaken through the University of Hong Kong to examine lumbar spine degeneration across a Chinese population [18]. Our sample size can be justified based on the functional approximation method proposed by Walter and Eliasziw [27]: Given n = 2 observations per rater, one-sided alpha = 0.05, beta = 0.20 (power = 0.8), an acceptable H(0) ICC of 0.75 and an expected ICC of 0.95 (based on previous research by Abbott et al. [19]), the computed acceptable sample size is n = 10. As such, the sample we use is appropriate for the reliability study. Image sets from three females and seven males aged 52.8 years (SD 7.2 years, range 44.0 to 60.8 years) with mixed imaging signs of disc degeneration and LBP history were selected. The two raters were blinded to all demographic and clinical details of the subjects. The over-arching prospective study and all associated investigations received ethics approval from the Institutional Review Board, Queen Mary Hospital, The University of Hong Kong, with written informed consent obtained from all participants.

### MRI measures and analysis

Two-dimensional single-echo axial T1-weighted MRI was achieved using a 3-Tesla MRI (Philips Healthcare, Best, The Netherlands). Parameters included: repetition time 500ms; echo time 9.5ms; rectangular field view, (74 %); thickness 4mm; flip angle 90°, and total acquisition duration 137 s. This scan included the caudal part of T12 to the cephalad portion of S3. Images were stored in DICOM format.

**Fig. 1:**
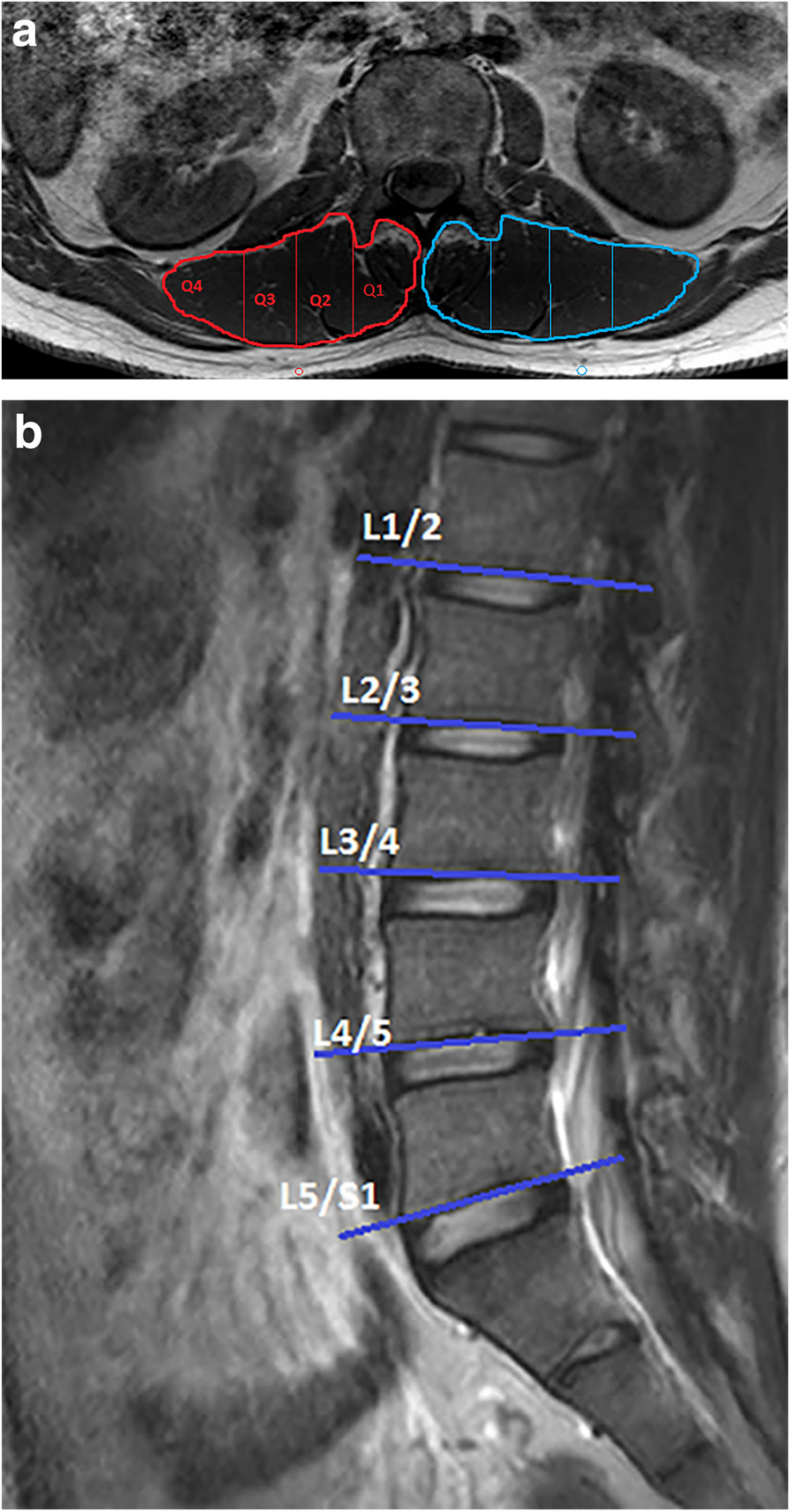
**a** Axial T1W MRI demonstrating bilateral paravertebral muscle regions of interest with simulated quartiled divisions, and bilateral referential (encircled) subcutaneous adipose tissue. **b** Sagittal T1W MRI indicating location of single slices used according to each lumbar level

A customized program was developed using MatLab (MathWorks, Inc, Natick, MA) to quantify the magnitude of MFI in each quartile of a defined region of interest (ROI) (Q1-4 (medial to lateral) and Q_mean_) based on muscle orientation as viewed in the axial plane. The program automatically derived quartiles based on pixel number within the ROI, where quartile 1 was most medial, and quartile 4 most lateral (simulated in Fig. 1a). MRI analysis consisted of manually-segmenting the ROI bilaterally encircling multifidus and erector spinae together (Fig. 1a). Mean pixel intensity from each ROI was reported as a percentage relative to a small encircled area of subcutaneous fat from the same level (Fig. 1a). Right, then left paravertebral group were outlined on a single-slice from the inferior vertebral end-plates of L1 through to L5 (Fig. 1b).

Images were segmented by two assessors: The first assessor (R1; *ANM*) had >250 h experience in lumbar paravertebral muscle fat content and volume analysis; the second (R2; *RJC*) had >1000 h. Both assessors initially had <10 h experience using the new method. Ten cases were measured twice, a week apart. Intra-rater agreement was determined from repeat measures of R1 and R2 combined, and inter-rater agreement comparing R1 with R2.

### Statistical analysis

Intra- and inter-rater reliability was determined using two-way mixed, absolute agreement intra-class correlation coefficients (ICC_3,1_) [28, 29] with corresponding 95 % confidence intervals (CI), and Bland-Altman plots including limits of agreement [30] that were used to assess the degree that two raters provided consistency of their individual ratings of overall FI, and FI according to lumbar level, quartile, and side. Several ICC cut-off values have been proposed to assess reliability [30, 31]. Following Portney and Watkins’ more rigid cut-off values for clinical measures, reliability was considered poor for ICCs <0.50, moderate for ICCs 0.50–0.75, good for ICCs 0.75–0.90, and excellent for values above 0.90 [31]. All statistical analyses were performed using Stata Version 14 (StataCorp, College Station, TX). For all analyses, the significance level was set to *p* ≤ 0.05.

## Results

**Fig. 2:**
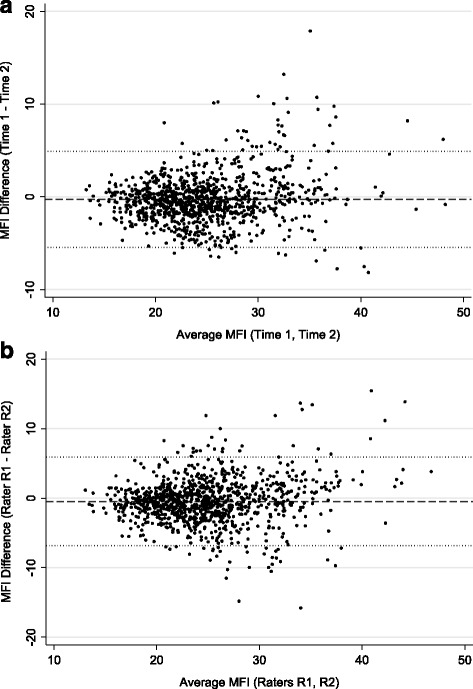
**a** Bland Altman plot for intra-rater reliability (where R1&R2 are combined). Footnote: Average muscle fatty infiltration (MFI %) of the two repeat observations (Time 1&2; x-axis), and the difference between MFI % values for each observation (*y*-axis). **b** Bland Altman plot for inter-rater reliability. Footnote: Average muscle fatty infiltration (MFI %) of the two raters (x-axis) versus the difference between MFI % values for each rater (y-axis)

Overall, Bland-Altman and ICC analyses showed high levels of agreement for intra-rater and inter-rater measures of MFI. The mean intra-rater difference (-0.28) and the corresponding limits of agreement (-5.48, 4.92) showed slightly better agreement than inter-rater agreement with an average difference of -0.48 (limits of agreement: -6.85, 5.90). Similarly, ICC for intra-rater reliability (ICC = 0.88; CI: 0.87–0.90) was slightly higher than intra-class correlation coefficients for inter-rater reliability (ICC = 0.82; CI: 0.80–0.84). With values above 0.80, intra-rater and inter-rater ICCs showed good levels of reliability. Furthermore, Bland-Altman plots showed no systematic association between FI values and absolute differences for either intra-rater (Fig. 2a) or inter-rater measures (Fig. 2b).

Intra-rater reliability results by lumbar level, quartile, and side are presented in Table 1 and inter-rater reliability in Table 2. Intra-rater reliability for all quartile average (Q_mean_) was highest at L5 (ICC = 0.89; CI: 0.79–0.94) and lowest at L1 (ICC = 0.61; CI: 0.37–0.78) and was higher for L1-5 at Q1 (ICC = 0.93; CI: 0.91–0.95) or Q2 (ICC = 0.89; CI: 0.86–0.92) than Q3 (ICC = 0.83; CI: 0.78–0.87) or Q4 (ICC = 0.81; CI: 0.76–0.85). Intra-rater reliability was better on the right (ICC = 0.91; CI: 0.90–0.93) than the left (ICC = 0.85; CI: 0.83–0.88).

**Table 1:** Intra-rater reliability (ICC; CI) according to lumbar level, quartile, and side

	L1	L2	L3	L4	L5	L1-5
Q1	0.84	0.85	0.86	0.89	0.94	0.93
	0.71–0.91	0.74–0.92	0.76–0.93	0.81–0.94	0.88–0.97	0.91–0.95
Q2	0.72	0.87	0.89	0.84	0.92	0.89
	0.38–0.86	0.76–0.93	0.81–0.94	0.72–0.91	0.85–0.97	0.86–0.92
Q3	0.71	0.70	0.81	0.76	0.88	0.83
	0.46–0.85	(0.51–0.83)	0.67–0.90	0.59–0.86	0.78–0.93	0.78–0.87
Q4	0.75	0.67	0.87	0.84	0.83	0.81
	0.53–0.87	0.45–0.81	0.75–0.93	0.72–0.91	0.70–0.91	0.76–0.85
Q_mean_	0.61	0.70	0.87	0.85	0.91	0.85
	0.37–0.78	0.50–0.83	0.77–0.93	0.74–0.92	0.84–0.95	0.81–0.89
Right	0.82	0.87	0.86	0.88	0.95	0.91
	0.74–0.87	0.81–0.91	0.79–0.90	0.83–0.92	0.93–0.97	0.90–0.93
Left	0.77	0.69	0.87	0.81	0.88	0.85
	0.59–0.87	0.54–0.80	0.81–0.91	0.73–0.87	0.83–0.92	0.83–0.88

Lumbar level (L1,L2,L3,L4,L5) and all lumbar levels combined (L1-5); Quartiles (Q1,Q2,Q3,Q4) where 1 is most medial, and 4 most lateral, and average of all 4 (Q_mean_)

**Table 2:** Inter-rater reliability (ICC; CI) according to lumbar level, quartile, and side

	L1	L2	L3	L4	L5	L1-5
Q1	0.79	0.78	0.85	0.86	0.87	0.90
	0.64–0.89	0.63–0.88	0.71–0.92	0.74–0.92	0.72–0.93	0.87–0.92
Q2	0.68	0.75	0.89	0.75	0.89	0.85
	0.47–0.82	0.57–0.86	0.79–0.94	0.57–0.86	0.79–0.94	0.81–0.88
Q3	0.63	0.43	0.61	0.70	0.87	0.75
	0.40–0.78	0.11–0.66	0.07–0.83	0.51–0.83	0.76–0.92	0.66–0.82
Q4	0.59	0.57	0.63	0.72	0.80	0.69
	0.34–0.76	0.32–0.74	0.11–0.83	0.50–0.85	0.65–0.89	0.57–0.76
Q_mean_	0.33	0.55	0.80	0.78	0.89	0.78
	0.03–0.58	0.30–0.74	0.41–0.92	0.63–0.88	0.79–0.94	0.72–0.83
Right	0.63	0.70	0.74	0.82	0.94	0.85
	0.47–0.75	0.58–0.79	0.52–0.85	0.75–0.88	0.91–0.96	0.82–0.87
Left	0.76	0.64	0.79	0.74	0.82	0.80
	0.66–0.83	0.50–0.74	0.45–0.90	0.63–0.85	0.74–0.88	0.76–0.83

Lumbar level (L1,L2,L3,L4,L5) and all lumbar levels combined (L1-5); Quartiles (Q1,Q2,Q3,Q4) where 1 is most medial, and 4 most lateral, and average of all 4 (Q_mean_)

Inter-rater reliability for Q_mean_ was also higher at L5 (ICC = 0.89; CI: 0.79–0.94), than L1 (ICC = 0.33; CI: 0.03–0.58) and L2 (ICC = 0.55; CI: 0.30–0.74), and higher for L1-5 at Q1 (ICC = 0.90; CI: 0.87–0.92) and Q2 (ICC = 0.85; CI: 0.81–0.88) than Q4 (ICC = 0.69; CI: 0.57–0.76). Inter-rater repeatability was good for both right (ICC = 0.85; CI: 0.82–0.87) and left (ICC = 0.80; CI: 0.76–0.83) sides.

## Discussion

Our investigation showed good intra- and inter-rater reliability for our method in quantifying the spatial distribution of lumbar paravertebral muscle fat content based on axial T1-weighted MRIs. Methodological implications were derived from our findings where lumbar level, intra-regional quartile, and side, was shown to influence repeatability.

Our results are an encouraging reflection of the clinical utility of this method that enables quantification of the spatial distribution of fat content in the lumbar paravertebral muscles. Using a comparable method for determining the geography of FI in the cervical spine based on multi-echo Dixon MRI, Abbott et al. [19] showed excellent intra-rater (ICC = 0.98; CI: 0.97–0.98) and inter-rater (ICC = 0.93; CI: 0.90–0.94) repeatability. Yet, attesting to the novelty of our method, no studies exist for direct comparison that determine quartiled MFI spatial distribution for the lumbar spine. The higher reliability reported by Abbott et al. for the cervical spine may relate to their use of fat-water-separated sequenced images, and/or morphological distinction between the spinal regions of interest.

Despite an increasing interest in quantifying MFI in the lumbar paravertebral muscles, surprisingly few studies report their methodological reliability, and instead focus on cross-sectional area and volume. Employing opposed-phase MRI to assess lumbar multifidus and erector spinae, Paalane and colleagues [26] report good intra-rater reliability with ICCs ranging from 0.86 to 0.88, and inter-rater values from 0.85 to 0.87. A tendency toward lower values for lumbar paravertebral muscle FI are described according to right (ICC = 0.82; CI: 0.16–0.96) and left (ICC = 0.78; CI: 0.12–0.95) sides by Valentin and colleagues [32] in assessing these muscles based on T1-weighted MRIs. In their study examining three multi-echo MRI sequencing techniques as contemporarily preferred for examining soft-aqueous tissues, Fischer and colleagues [10] describe good to excellent inter-rater agreement ranging between ICC = 0.84–0.90; they did not report intra-rater values. As such, the overall repeatability of our method appears acceptable.

We showed highest reliability at L5 and lowest at L1 or L2, which probably relates to ease of identification wherein lower lumbar levels have higher FI in multifidus and erector spinae [13], and may have a more defined morphology distinguishable from adjacent structures. Unfortunately no other studies provide analysis to corroborate this claim. Reliability tended to be higher medially (Q1&2) than laterally (Q3&4); we speculate that this again relates to distinction between morphology where the two medial quartiles are bordered by the vertebral landmarks between the spinous and transverse processes and are therefore more easily delineated. An interesting finding from Valentin and colleagues [32] indicated that multifidus required more experience of the rater to achieve an acceptable repeatability than the other paravertebral muscles they examined (including erector spinae). As multifidus is the most medial and deep of the lumbar extensor group abutting boney landmarks, our speculation appears to contradict their finding.

Repeatability was higher for the right compared to the left. We speculate this may have a methodological basis where we commenced each case on the right side; to eliminate any likelihood for this bias, we propose that future studies employing this or other skeletal muscle FI quantification methods should randomize the starting side. In the only other study publishing reliability metrics, Valentin and colleagues [32] showed variable ICCs according to individual muscle and side. While we describe values based on a single slice per lumbar level, Valentin and colleagues [32] report volume for each muscle over multiple levels. Confidence intervals for both raters in our study are generally narrower than theirs, which may relate to different methods, but is an encouraging reflection of the repeatability of our method.

The results of our study should be interpreted in consideration of its limitations. While not the central focus of this technical study, the small sample used make it difficult to draw conclusions regarding the relevance of the spatial distribution of fat content in lumbar paravertebral muscles. Only additional studies examining various clinical groups will establish whether there is merit in pursuing this new direction. However, there was sufficient power in the ten cases for a reliability assessment of ICCs, and as such we delivered on our aim in establishing the reliability of our method.

## Conclusions

We present a reliable method for determining the spatial distribution in the transverse plane of fat content in the lumbar paravertebral muscles based on conventional T1-weighted MRI. Application of this method to large population-based datasets may advance the field’s understanding of the contribution of paravertebral muscle quality to spine health, and allow for identification of where best to direct interventions.

## Abbreviations

CI, 95 % confidence intervals; FI, Fatty infiltration; L1,2,3,4,5, Lumbar levels L1 to L5; LBP, Low back pain; MRI, Magnetic resonance imaging; Q1-4, Quartiles 1 (medial) to 4 (lateral); R1/R2, Rater 1/Rater 2; T1-W, T1-weighted.
